# Evolution of three Nobel Prize themes and a Nobel snub theme in chemistry: a bibliometric study with focus on international collaboration

**DOI:** 10.1007/s11192-017-2377-z

**Published:** 2017-04-26

**Authors:** Sichao Tong, Per Ahlgren

**Affiliations:** 1 National Science Library, Chinese Academy of Sciences, Beijing, 100190 China; 2 School of Education and Communication in Engineering Sciences (ECE), KTH Royal Institute of Technology, 100 44 Stockholm, Sweden; 3 University of Chinese Academy of Sciences, Beijing, 100049 China

**Keywords:** Bibliometrics, Chemistry, Evolution, International collaboration, Nobel Prize, Publication volume

## Abstract

In this study, three chemistry research themes closely associated with the Nobel Prize are bibliometrically analyzed—Ribozyme, Ozone and Fullerene—as well as a research theme in chemistry not associated with the Nobel Prize (a Nobel snub theme): Brunauer–Emmett–Teller equation. We analyze, based on an algorithmically constructed publication-level classification system, the evolution of the four themes with respect to publication volume and international collaboration, using two datasets, one of them a subset of highly cited publications, for each considered time period. The focus of the study is on international collaboration, where co-occurrence of country names in publications is used as a proxy for international collaboration. For all four themes, especially for Brunauer–Emmett–Teller equation, the publication volumes increase considerably from the earliest period to the later periods. The international collaboration rate shows an increasing trend for each theme. For Ozone, Fullerene and Brunauer–Emmett–Teller equation, the international collaboration rate tend to be higher for the highly cited publications compared to full datasets. With regard to the evolution of number of countries per international publication and per highly cited international publication, a vast majority of the distributions are positively skewed, with a large share of publications with two countries. With respect to the last four periods of the study, the concentration to two countries per publication is more pronounced for the Brunauer–Emmett–Teller equation theme compared to the three Nobel Prize themes.

## Introduction

The scientific Nobel Prizes receive much attention, not only within the scientific community, and might be regarded as the ultimate accolades in science (Merton 1968). A number of studies have utilized bibliometric methods in relation to the scientific Nobel Prizes. In a work by Gingras and Wallace (2010), rankings of Nobel laureates, and of other nominees, based on citation impact and degree centrality in author co-citation networks were analyzed. Rodriguez-Navarro (2011) proposed a bibliometric indicator based on the number of highly cited publications and observed that the indicator was (on the country level) highly correlated with the number of Nobel Prize achievements. Heinze et al. (2013) used two Nobel Prize-winning contributions, the scanning tunneling microscope and the discovery of fullerene, and examined the growth of follow-up research, in terms number of citing publications, at the author and sub discipline level. Bjork et al. (2014) analyzed the citation trajectories of Nobel laureates in economics, whereas Wagner et al. (2015) compared Nobel laureates in physiology or medicine to a matched group of scientists in order to examine productivity, impact, co-authorship and international collaboration patterns within research networks. Chan et al. (2015) studied co-authorship patterns before and after Nobel Prize reception, and the same authors analyzed interactions of co-authorship and scientific productivity in Nobel laureate teams (Chan et al. 2016). Recently, Hu and Rousseau (2017), using publications by 2016 Nobel Prize laureates, proposed new citation-based indicators to recognize foundational work in science.

As stated by Bruckner et al. (1990), citation analysis, text analysis and scientometric indicators, among other methods, help to understand scientific evolution. Boyack et al. (2009) mapped the structure and evolution of chemistry research, based on a journal co-citation analysis. Neff and Corley (2009) used co-word analysis to study the evolution of ecology during the period 1970–2005, while the evolution of sleep science was analyzed by Robert et al. (2007). An approach, involving, bibliometric indicators, to analyze the thematic evolution of a research field was presented by Cobo et al. (2011), and the fuzzy set theory field was used as an example. Cantos-Mateos et al. (2012) dealt with the evolution of Spain’s scientific output in stem cells research during the period 1997–2007, and Liu et al. (2012) explored the evolution of earthquake research over a large time period and, in particular, analyzed the evolution of the most frequently used keywords. The evolution of scientific topics has recently been analyzed (e.g., Jensen et al. 2016; Mryglod et al. 2016), as well as the evolution co-publishing networks in Korea (Kim et al. 2016).

In this study, three chemistry research themes closely associated with the Nobel Prize are bibliometrically analyzed—Ribozyme, Ozone and Fullerene—as well as a research theme in chemistry not associated with the Nobel Prize but a Nobel snub theme: Brunauer–Emmett–Teller equation (BET equation). The purpose of the study is analyze the evolution of the four themes with respect publication volume and international collaboration. In particular, we aim at comparing the three Nobel Prize themes with the BET equation theme with respect to potential evolution differences. For instance, one might ask if the evolution is driven by the Nobel Prize or not. To shed light on such questions, it is important to include at least one (central) non-Noble Prize theme in the study.

In order to fulfill the purpose of the study, an automatically constructed, hierarchical publication-level classification system and two datasets are used. The focus of the study is on international collaboration, where co-occurrence of country names in publications is used as a proxy for international collaboration. We believe it is useful study international collaboration in view of its potential important influences on the evolution of scientific research. Indeed, international collaboration, considered as a common and functional characteristic in the production and development of research, has been increasingly analyzed, varying from the analysis of its patterns to designing policies for supporting research (e.g., Beaver and Rosen 1978; Teasley and Wolinsky 2001; Wuchty et al. 2007).

The remainder of this paper is organized as follows. In the next section, we describe the data and methods of the study. The results of the study are given in the third section, whereas the fourth section contains a discussion and put forward conclusions.

## Data and methods

The official web site of the Nobel Prize was explored in order to select three chemistry themes associated with the prize.^1^ To obtain an important chemistry theme, which has not been awarded the Nobel Prize, an article in *Chemical and Engineering News* on notable Nobel snubs was examined.^2^ The following four themes were selected, where the three Nobel Prize themes are listed first (the year inside parenthesis concerns the year in which the prize was awarded):

- Ribozyme: The discovery of catalytic properties of RNA (1989).
- Ozone: The work in atmospheric chemistry, particularly concerning the formation and decomposition of ozone (1995).
- Fullerene: The discovery of fullerenes (1996).^3^
- BET equation, named after its three inventors (Stephen Brunauer, Paul Hugh Emmett, and Edward Teller): The equation results from BET theory, which aims to explain the physical adsorption of gas molecules on a solid surface.

The Nobel laureate lectures corresponding to the three themes (a)–(c) appear as publications in Web of Science, and these publications were retrieved and used as seed publications. For BET equation, we obviously had to take another approach. We did a topic search^4^ on BET equation in Web of Science, and ordered the retrieved publications descending after received citations. The most frequently cited publication was used as a seed publication for the BET equation theme. For each of the four seed publications, additional publications were retrieved with the aid of the Web of Science tool “View Related Records”. This tool retrieves publications that are bibliographically coupled to a given publication, i.e., publications that share at least one cited reference with the given publication. For each of the seed publications, the three publications, of the Web of Science document types *Article* or *Review*, with the largest number of shared references (with the seed publication) were recorded.^5^

In order to obtain fuller sets of publications related to the four themes in chemistry, we used Bibmet, the in-house version of Web of Science at KTH Royal Institute of Technology, Stockholm, Sweden. Bibmet contains publications published between 1980 and 2016. Based on a methodology put forward by Waltman and van Eck (2012, 2013), the bibliometric group at KTH has implemented a hierarchical publication-level classification system. About 28 million Bibmet publications of the Web of Science document types *Article* or *Review* have been algorithmically grouped into classes based on direct citation links between them. The classification consists of four hierarchical levels: level 4 with 22 broad classes, level 3 with 673 classes, level 2 with 3935 classes, and level 1 with 35,192 classes. For each class in the system, content labels have been obtained automatically. The terms used as content labels are keywords, journal names, names of Web of Science journal categories, and titles of review articles. For each class, the three most relevant terms, where relevance is based on the tf-idf scheme (Manning et al. 2008), were selected as content labels.^6^

The class (or classes) at level 1 to which the three related publications, for a given seed publication, belong was identified. However, the number of publications of the identified classes turned out to be too small for a meaningful analysis. Therefore, for a given seed publication, the class at level 2 of the three related publications was identified.^7^ The content labels of the four identified level 2 classes are the following, where the labels are separated by “//”:

- RIBOZYME//GROUP I INTRON//RIBOSWITCH
- JOURNAL OF GEOPHYSICALRESEARCH-ATMOSPHERES//MIDDLEATMOSPHERE DYNAMICS//MESOSPHERE
- FULLERENES//FULLERENE//FULLERENE SCIENCE AND TECHNOLOGY
- MESOPOROUS MATERIALS//MESOPOROUS SILICA//MICROPOROUS AND MESOPOROUS MATERIALS

Note that (a), (b), (c) and (d) in this list corresponds to (a), (b), (c) and (d), respectively, in the first list of this section. In the rest of this work, we denote the class associated with (a) by Ribozyme_c, with (b) by Ozone_c, with (c) by Fullerene_c and by (d) by BET_c. Further, we refer to the four classes by the term “research fields”.

For the analysis of the evolution over time, with respect to publication volume and international collaboration, of the four research fields, the publication period of the study, 1980–2015, was divided into the following 3-year periods: T1 = 1980–1982, T2 = 1983–1985, T3 = 1986–1988, T4 = 1989–1991, T5 = 1992–1994, T6 = 1995–1997, T7 = 1998–2000, T8 = 2001–2003, T9 = 2004–2006, T10 = 2007–2009, T11 = 2010–2012, and T12 = 2013–2015.

For each research field and each time period, we considered two datasets: the set of publications in the research field published during the time period, say *S*, and the subset of *S* of its highly cited publications. We define a *highly cited publication* as a publication belonging to the 20% most frequently cited publications in *S*.

The indicators used in the study can be divided into two categories: indicators based on publication volume, and indicators based on country (name) volume within publications. The following indicators are based on publication volume:

1. P: Number of publications.
2. IP: Number of *international publications*, defined as publications co-authored by two or more countries.
3. Ph: Number of highly cited publications.
4. IPh: Number of international publications among the highly cited publications.
5. IPR: Percentage of IP relative to P.
6. IPRh: Percentage of IPh relative to Ph.
7. ΔIPR(Tn): Degree of alteration of IPR at Tn relative to T(n − 1) (ΔIPR(Tn) = | IPR(Tn) − IPR(T(n − 1))|, 1 < n ≤ 12).
8. ΔIPRh(Tn): Degree of alteration of IPRh at Tn relative to T(n − 1) (ΔIPRh(Tn) = | IPRh(Tn) − IPRh(T(n − 1))|, 1 < n ≤ 12).

We collectively refer to the indicators 5 and 6 in the list above as “International collaboration rate”. The alteration indicators 7 and 8, inspired by Sangwal (2015), facilitate the identification of abrupt changes in international collaboration rate. The following indicators are based on country volume within publications:

9. CIP: Number of countries per international publication.
10. CIPh: Number of countries per highly cited international publication.

### Remark on the selection of themes

Admittedly, the selection of the four themes, especially the three Nobel Prize themes, is quite arbitrary. However, as indicated above, Bibmet has 1980 as the earliest publication year. In view of this, we decided to select Nobel Prize themes that were awarded the prize no earlier than approximately 1990. Obviously, for a theme to be selected it should not have been awarded the prize too late. Therefore, it was decided to select themes that were awarded the prize no later than approximately 2005. A further condition was that for each of the four seed publications, its three related publications should belong to the same class at level 2 of the Bibmet publication-level classification system (cf. footnote 7). If a theme does not satisfy this condition, the union of two or three classes can be used. However, such a union might involve a lot of publications that are not connected, or weakly connected, to the theme. Expressed in another way, the precision of the union might be low. Several initially considered chemistry Nobel Prize themes did not satisfy the condition.

## Results

Table 1 reports number of publications and international publications by research field and time period, whereas Table 2 reports corresponding numbers for highly cited publications and international highly cited publications. For instance, for Table 1, the number of publications (P) of the research field Ozone_c in time period T2 is equal to 1097. We concentrate on the evolution of P. Note that the number of publications decreases from T11 to T12 for each research field except BET_c. This outcome is explained by an incomplete set of publications from the last considered publication year of the study, 2015. When the data collection was done, a considerable amount of Web of Science articles/reviews from 2015 was missing in the classification system of Bibmet. This limitation should be kept in mind when the publication volume results are interpreted.

**Table 1:** Number of publications (P) and international publications (IP) by research field and time period

Time period	Ribozyme_c		Ozone_c		Fullerene_c		BET_c	
	IP	P	IP	P	IP	P	IP	P
T1	6	68	75	930	2	30	0	6
T2	14	131	108	1097	6	37	1	8
T3	38	238	183	1156	5	99	0	8
T4	62	405	190	1324	59	425	1	16
T5	85	622	337	1691	451	2796	15	96
T6	95	880	510	2000	679	3405	71	442
T7	135	941	541	1891	733	2903	172	875
T8	148	983	679	2109	762	2547	336	1550
T9	212	1136	829	2192	755	2510	451	2294
T10	233	1304	876	2227	681	2404	492	2581
T11	303	1419	855	2218	685	2227	629	3103
T12	281	1267	788	1960	561	1851	649	3148
Total	1612	9394	5971	20,795	5379	21,234	2817	14,127

**Table 2:** Number of highly cited publications (Ph) and international highly cited publications (IPh) by research field and time period

	Ribozyme_c		Ozone_c		Fullerene_c		BET_c	
Time period	IPh	Ph	IPh	Ph	IPh	Ph	IPh	Ph
T1	2	14	14	186	1	6	0	1
T2	2	26	21	219	1	7	0	2
T3	10	48	44	231	1	20	0	2
T4	10	81	41	265	10	85	0	3
T5	16	124	63	338	87	559	4	19
T6	17	176	101	400	153	681	12	88
T7	25	188	123	378	182	581	43	175
T8	37	197	136	422	188	509	74	310
T9	41	227	218	438	191	502	103	459
T10	50	261	238	445	161	481	98	516
T11	70	284	226	444	206	445	143	621
T12	54	253	202	392	136	370	145	630
Total	334	1879	1427	4158	1317	4246	622	2825

The number of publications in the Ribozyme_c field increases strictly from T1 to T11, from 68 publications in T1 to 1419 in T11 (it follows that also the number of highly cited publications (Ph) increases strictly, since these constitute 20% of the number of publications for each period). This yields that the publication volume is about 21 times higher in T11 compared to T1. In the Ozone_c field, we observe a strict increase in publication volume from T1 to T6, from 930 to 2000 publications. The peak occurs in the period T10 (2227 publications, about 2.4 times the volume in T1). From T6 onwards, the number of publications is relatively stable. In the Fullerene_c field, publication volumes are relatively small up to the period T4. A steep increase occurs from T4 to T6, which is the period of the volume peak (3405 publications, about 113.5 times the volume in T1). From T6, the publication volume strictly decreases. Ozone_c has much larger publication volumes in initial periods than Ribozyme_c and Fullerene_c, and Ozone_c and Fullerene_c have total publications volumes more than twice the corresponding volume of Ribozyme_c (the last row of Table 1).

The field BET_c has, compared to the three Noble Prize fields, few publications in the first six periods. In T1–T4, the maximum number of publications is 16, and consequently, the maximum number of highly cited publications is also low for these periods (Table 2, the rightmost column). However, as is clear from Table 1, a steep increase occurs from T5 to T11. Indeed, from T10 onwards, BET_c has the highest publication volumes of the four research fields.

**Fig. 1:**
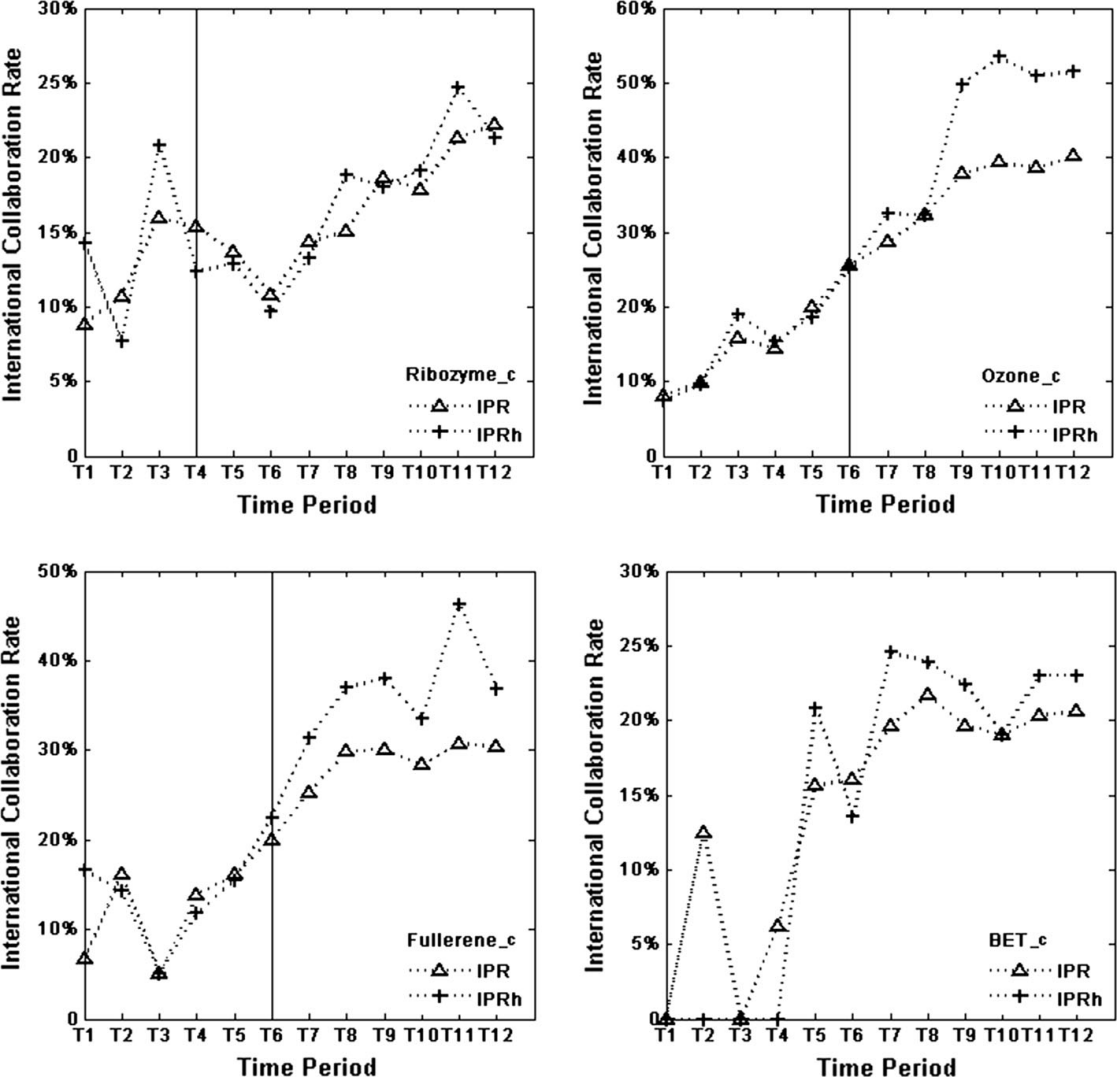
IPR and IPRh over time for the four research fields. Vertical lines in the sub charts of the Nobel Prize themes indicate the time period in which the prize was awarded

Figure 1 shows the evolution of IPR (percentage of IP relative to P) and IPRh (percentage of IPh relative to Ph) for the three research fields. Regarding Ribozyme_c, there is an increasing trend for IPR and IPRh from T6 onwards, even if the international collaboration rate decreases from the next last to the last period for the highly cited publications. From T4—the period in which the Nobel Prize was awarded to the Ribozyme theme—to T6, a decreasing trend is observed for IPR. In the Ozone_c case, an increasing trend from the first considered period, for both IPR and IPRh, is observed, with the maximum IPR (IPRh) value in T12 (T10). Note the large increase from T8 to T9 for IPRh (from T9, the international collaboration rate is quite stable). In Fullerene_c, IPR shows an increasing trend from T3. The same holds for IPRh up to T9. In Ozone_c and Fullerene_c, the curves for IPRh tend to appear above the curves for IPR, which means that the international collaboration rate tend to be higher for the highly cited publications for these two research fields. This trend is more pronounced in the periods that follow the period of the award (T6, which is marked with a vertical line in the two corresponding sub charts). The trend can be observed also for the snub field BET_c with respect to T7 onwards. Across T1–T6, the two curves are highly irregular.

**Fig. 2:**
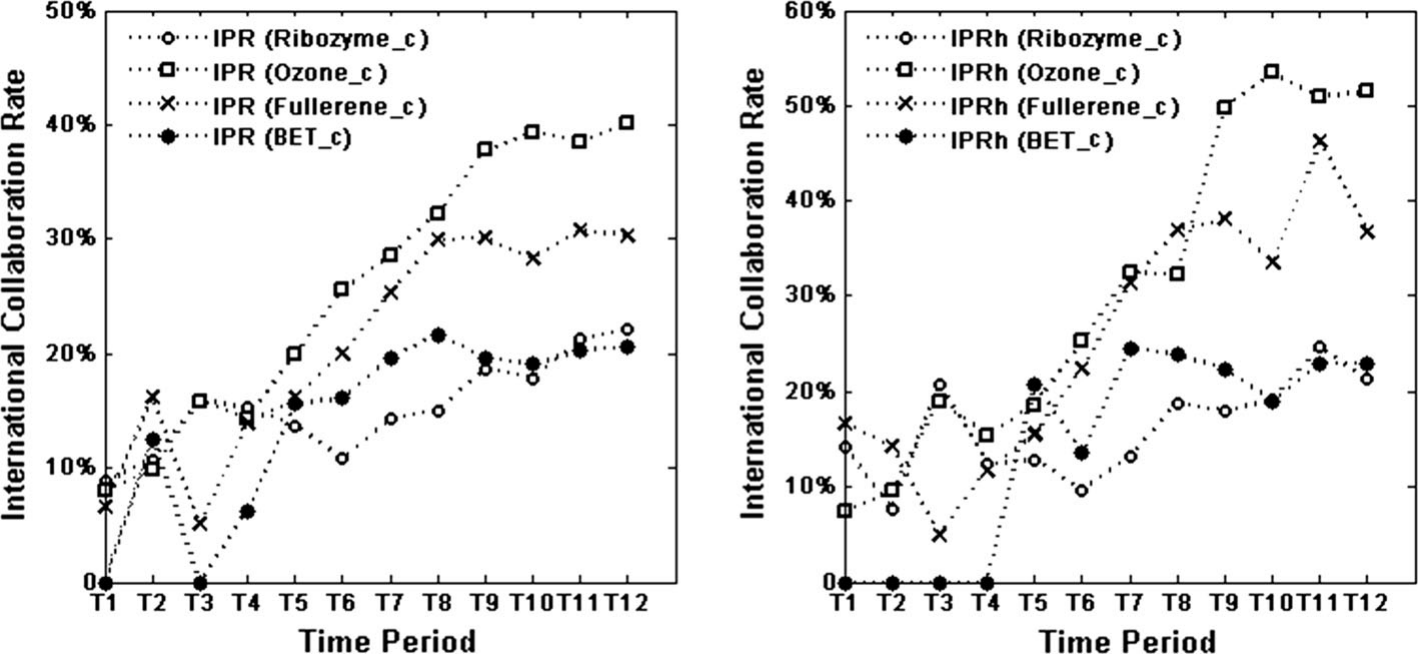
IPR and IPRh over time: comparison between the four research fields

Figure 2 has the same underlying data as Fig. 1. However, here we focus on comparison between the four research fields. Generally, and irrespective of dataset, Ozone_c tend to have the highest international collaboration rate, followed by Fullerene_c. The international collaboration rates for these two fields are for several periods quite similar, whereas the rates for Ribozyme_c and BET_c, from T6 onwards, are considerably lower, regardless of dataset.

**Fig. 3:**
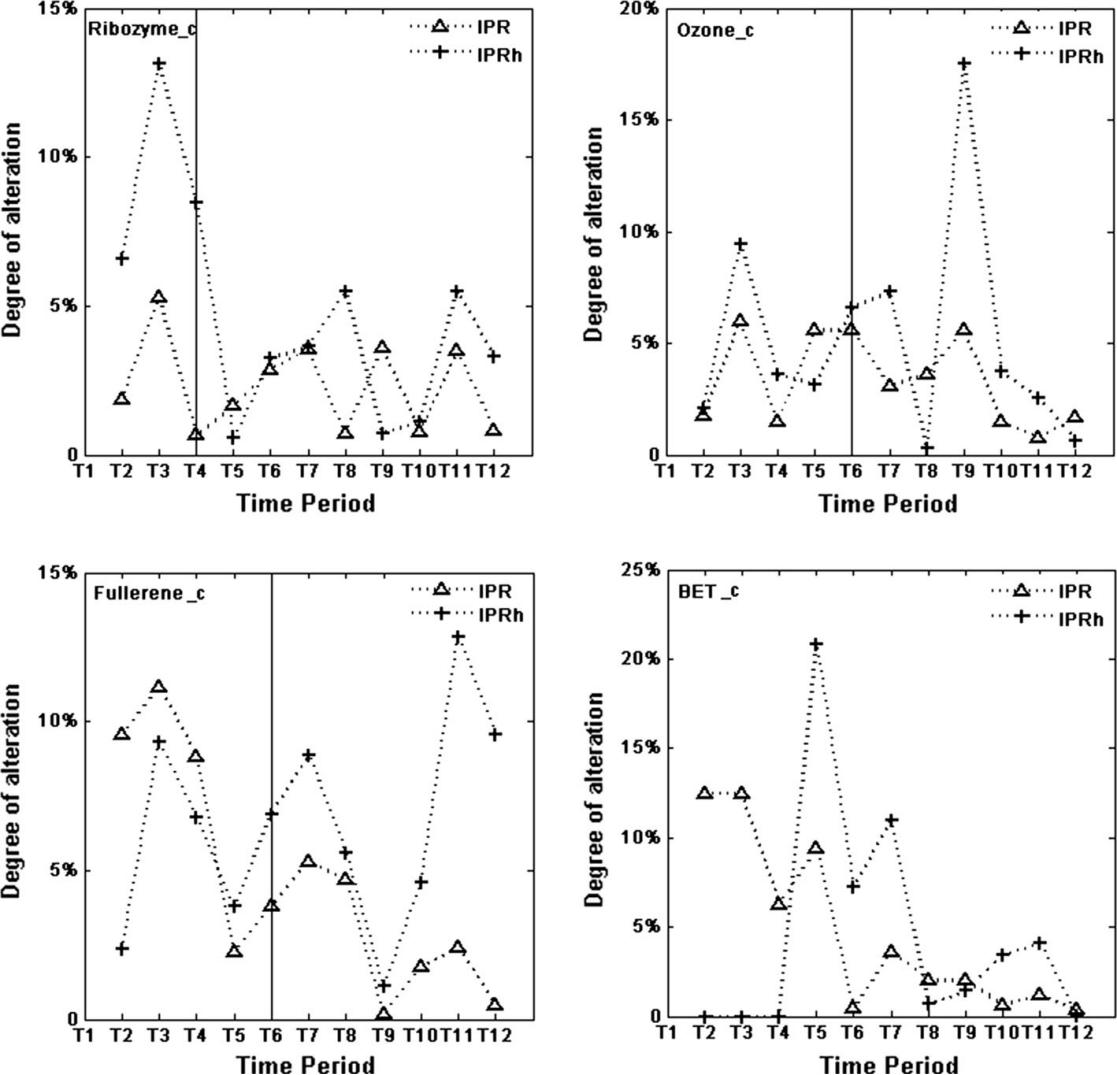
ΔIPR(Tn) and ΔIPRh(Tn), 1 < n ≤ 12, for the four research fields

Figure 3 concerns degree of alteration. In the Ribozyme_c research field, from T2 up to T4, IPRh has higher degrees of alteration than IPR. One should keep in mind, however, that the number of highly cited publications is fairly low in the first four periods for this field (Table 1). Also for the last two time periods, IPRh has higher degrees of alteration than IPR. In the Ozone_c field, IPRh has higher degrees of alteration than IPR except for the periods T5, T8 and T12. The abrupt change from T8 to T9 for IPRh, indicated above, is clearly visible in the chart for Ozone_c. The degree of alteration at T9 is 17.5%. With regard to Fullerene_c, and from T5 onwards, IPRh has higher degrees of alteration than IPR, in particular in the last two periods. The maximum alteration degree, regardless of research field and dataset, concerns BET_c (IPRh at T5). However, the number of highly cited publications in T4 and T5 are only 3 and 19, respectively (Table 2).

**Fig. 4:**
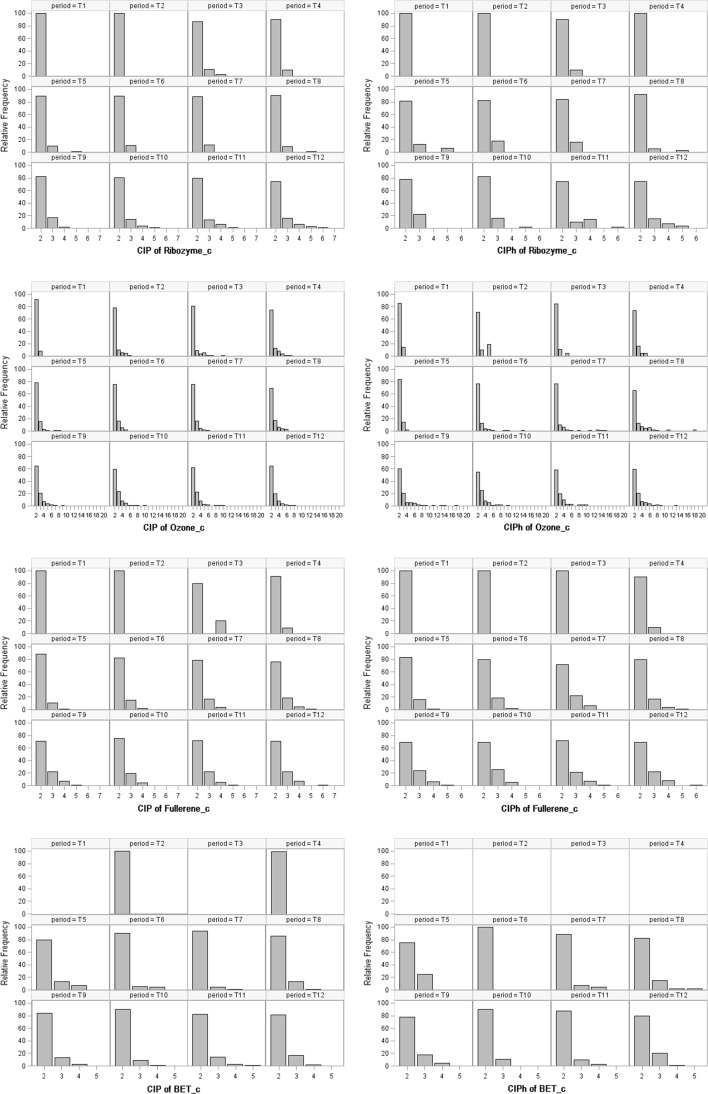
Relative frequency distributions for CIP and CIPh over time for the four research fields

Now we turn our attention to the evolution of the number of countries per international publication and per highly cited international publication, i.e., to the evolution of CIP and CIPh. Here we deal with, per research field and time period, two publication sets: the set of international publications, and the set of highly cited international publications. Figure 4 displays relative frequency distributions for the four research fields Ribozyme_c, Ozone_c Fullerene_c and BET_c. For instance, with regard to Ribozyme_c, the indicator CIPh and the period T9, the share of publications with three countries is about 22% (9 of 41; Appendix, Table 4). The number of international publications is very small for some of the earlier time periods, in particular for BET_c and Fullerene_c (Appendix, Tables 3, 4, 5, 6, 7, 8, 9, 10). Therefore, we concentrate on the later periods.

**Table 3:** Frequency distributions of CIP for Ribozyme_c

	T1	T2	T3	T4	T5	T6	T7	T8	T9	T10	T11	T12
2	6	14	33	56	76	85	120	134	174	187	241	209
3			4	6	8	10	15	13	35	33	39	45
4			1						3	9	19	18
5					1			1		3	3	7
6											1	2
7										1		
Total	6	14	38	62	85	95	135	148	212	233	303	281

**Table 4:** Frequency distributions of CIPh for Ribozyme_c

	T1	T2	T3	T4	T5	T6	T7	T8	T9	T10	T11	T12
2	2	2	9	10	13	14	21	34	32	41	52	40
3			1		2	3	4	2	9	8	7	8
4											10	4
5					1			1		1		2
6											1	
Total	2	2	10	10	16	17	25	37	41	50	70	54

**Table 5:** Frequency distributions of CIP for Ozone_c

	T1	T2	T3	T4	T5	T6	T7	T8	T9	T10	T11	T12
2	69	85	148	142	264	388	408	470	537	517	529	510
3	6	11	16	1	52	82	88	114	169	209	194	154
4		6	6	1	10	26	22	41	62	72	71	62
5		5	10	15	3	7	12	26	28	39	23	31
6		1	1	7	1	1	4	16	16	8	15	13
7			1	24	4	2		3	4	7	3	7
8					2	1	1	2	1	9	6	7
9						1	1		4	4	5	1
10			1		1	1				6	7	
11							1	3	2	2		1
12										1	1	1
13							2	1	1			1
14						1	1		3	1	1	
15							1	1	1			
17									1			
18								2				
19										1		
Total	75	108	183	190	337	510	541	679	829	876	855	788

**Table 6:** Frequency distributions of CIPh for Ozone_c

	T1	T2	T3	T4	T5	T6	T7	T8	T9	T10	T11	T12
2	12	15	38	30	54	79	93	91	130	131	132	120
3	2	2	4	7	8	12	12	17	44	62	44	42
4				2	1	3	9	8	12	20	23	16
5		4	2	2		3	2	5	12	12	7	12
6						1	1	7	9	2	5	5
7								2	4	3	1	2
8							1	1	1	4	5	3
9						1			2		5	1
10						1				2	3	
11							1	3	1			
12										1	1	
13							2		1			1
14						1	1		1			
15							1					
17									1			
18								2				
19										1		
Total	14	21	44	41	63	101	123	136	218	238	226	202

**Table 7:** Frequency distributions of CIP for Fullerene_c

	T1	T2	T3	T4	T5	T6	T7	T8	T9	T10	T11	T12
2	2	6	4	54	397	558	580	582	534	511	490	395
3				5	49	104	121	142	164	132	151	122
4			1		5	15	29	33	52	33	38	39
5						2	2	5	5	3	5	2
6										2	1	3
7							1					
Total	2	6	5	59	451	679	733	762	755	681	685	561

**Table 8:** Frequency distributions of CIPh for Fullerene_c

	T1	T2	T3	T4	T5	T6	T7	T8	T9	T10	T11	T12
2	1	1	1	9	72	122	131	150	132	111	147	94
3				1	14	28	40	31	46	41	43	30
4					1	3	11	6	12	9	15	11
5								1	1		1	
6												1
Total	1	1	1	10	87	153	182	188	191	161	206	136

**Table 9:** Frequency distributions of CIP for BET_c

	T1	T2	T3	T4	T5	T6	T7	T8	T9	T10	T11	T12
2		1		1	12	64	162	287	380	442	519	528
3					2	4	8	46	60	44	90	107
4					1	3	2	2	11	5	17	13
5								1		1	3	1
Total	0	1	0	1	15	71	172	336	451	492	629	649

**Table 10:** Frequency distributions of CIPh for BET_c

	T1	T2	T3	T4	T5	T6	T7	T8	T9	T10	T11	T12
2					3	12	38	61	80	88	125	115
3					1		3	11	18	10	14	29
4							2	1	5		4	1
5								1				
Total	0	0	0	0	4	12	43	74	103	98	143	145

For Ribozyme_c, and for both publication sets, all distributions (with one exception) from the period T3 onwards are positively skewed, with a large share of publications with two countries. However, this share, which generally is above 80%, is 74% (the minimum value for the field regarding two countries) in T12 for both sets. In this period, we observe small shares of publications with five or six countries (the latter value only for CIP, though). With respect to Ozone_c, for both publication sets, all distributions are positively skewed, with a large share of publications with two countries. In the last four periods, however, the distributions are less concentrated, and publications with more than six countries can be observed. However, the absolute frequencies of such publications are small (Appendix, Tables 5 and 6). The minimum values for this field regarding two countries are 59% (T10; the set of international publications) and 55% (T10; the set of highly cited international publications). For Fullerene_c, and for both publication sets, a vast majority of the distributions are positively skewed, and again with a large share of publications with two countries. In the last four periods, the concentration to two countries per publications is less than in earlier periods. The minimum values for this field regarding two countries are about 70% (T12; the set of international publications) and 69% (T10; the set of highly cited international publications). Also for the snub field, BET_c, the distributions are generally positively skewed. With respect to the last four time periods, the concentration to two countries per publication is more pronounced for BET_c compared to the three Nobel Prize fields.

## Discussion and conclusions

In this bibliometric study, we have analyzed three chemistry research themes closely associated with the Nobel Prize—namely Ribozyme, Ozone and Fullerene—as well as a Nobel snub theme, BET equation. Based on an algorithmically constructed publication-level classification system, we analyzed the evolution of the four themes with respect to publication volume and international collaboration using two datasets, one of them a subset of highly cited publications, for each considered time period. For all four themes, we observed increasing publication volumes from the earliest period to the later periods, as well as an increasing international collaboration rate trend. For Ozone and Fullerene, the international collaboration rate tended to be higher for the highly cited publications compared to full datasets. This trend turned out to be more pronounced in the periods that follow the period of the award. With regard to the evolution of number of countries per international publication and per highly cited international publication, a vast majority of the distributions are positively skewed, with a large share of publications with two involved countries. Regarding the later periods and the three Noble Prize themes, though, the distributions turned out to be less concentrated. For BET_c with respect to the last four time periods, the concentration to two countries per publication is more pronounced compared to the three Nobel Prize themes.

An interesting question is whether the Nobel Prize is a signal that the main work in the corresponding research theme is done or that it generates credibility and followers to further push the theme. Regarding research field Ribozyme_c, publication volume strictly increases from the period T4, the period in which the prize was awarded to the corresponding theme, to the next last period. This suggests that the prize gave rise to followers that further pushed the Ribozyme theme. For Fullerene_c, however, publication volume strictly decreases from T6, the period in which the prize was awarded to the corresponding theme, to the next last period. This outcome suggests that the main work was done when the prize was awarded to the theme. A similar conclusion might be drawn for the Ozone theme: in Ozone_c, publication volume is fairly stable from T6, the period in which the prize was awarded to the theme, to the next last period.

It is clear that, generally, the results for the snub research field BET_c are similar to the results for the three Nobel Prize fields Ribozyme_c, Ozone_c and Fullerene_c. However, the publication volume growth rate for BET_c with respect to the periods T9 and T11, about 35%, is substantially higher compared to Ribozyme_c and Ozone_c (for Fullerene_c, a decrease is observed, as indicated in the preceding paragraph).^8^ Regarding the question, indicated in the section “Introduction”, whether the evolution is driven by the Nobel Prize or not, the results of our study do not support an affirmative answer, in view of the observed evolution similarities between, on the one hand, the three Nobel Prize themes and, on the other hand, the snub theme BET_c.

According to Cech (2002), the concept of ribozyme expanded already 1–2 years after 1982, the year in which the first catalytic ribozyme was reported. Such expansions might give rise to entire research fields, as seem to be the case for the Ribozyme theme. We believe that the expansion of the ribozyme concept is reflected in the substantial increase in (total) number of publications from period T1 (1980–1982) to period T2 (1983–1985) for the research field Ribozyme_c. With regard to the field Fullerene_c, the outcomes that publication volumes are relatively small up to the period T4, and that a steep increase occurs from T4 (1989–1991) to T5 (1992–1994), have an interesting counterpart in the study by Heinze et al. (2013). One of the outcomes of that study was that the number of the follow-up works (in terms of number of citing publications) of the fullerene breakthrough publication by Kroto et al. (1985) increased dramatically from 1990 to 1992.

Our results show that the international collaboration rate tends to be higher for the highly cited publications regarding the research fields Ozone_c, Fullerene_c and BET_c, but not regarding the field Ribozyme_c. The latter was quite unexpected in view of earlier research on the relationship between international collaboration and citation rates (e.g., Aksnes 2003; Smith et al. 2014), even if international collaboration not always yields higher citation rates (Rousseau and Ding 2016; Sud and Thelwall 2016).

We have to point out four limitations of our study. First, to obtain a deeper understanding of the evolution of the themes of the study, we believe it is proper to go further back in time than to the year 1980. However, we were not able to do that, since the earliest publication year of Bibmet is 1980. Second, the number of publications is low in the earlier periods for the research fields Ribozyme_c, Fullerene_c and BET_c. The results that concern these periods and fields should therefore be interpreted with caution. Third, the three algorithmically constructed fields used in the study cannot be assumed to be perfect representations of the three corresponding research themes. Some publications that clearly belong to the Ribozyme theme, for instance, might be missing in the field Ribozyme_c, whereas some publications that are only weakly connected to the theme might be present in Ribozyme_c. Forth, we are not chemists, which makes it difficult for us to put forward causes for the observed differences. Our understanding of these are, then, quite restricted. The use of subject experts as result interpreters is an interesting possibility in this respect, and we might realize this possibility in future, and similar, studies.

For future research, we would like to further explore how the algorithmically constructed publication-level classification system employed in this study can be used for the analysis and mapping of research themes. For a given theme, an alternative starting point might be a comprehensive review article with several cited references. The classes of the system to which the cited references, covered by Web of Science, belong can be identified for each hierarchical level of the system. By this, publication classes strongly connected to the theme might be obtained.

## Appendix

Frequency distributions for the four research fields: number of countries per international publication and per highly cited international publication. See Tables 3, 4, 5, 6, 7, 8, 9 and 10.

## Footnotes

1
https://www.nobelprize.org/.

2
http://cen.acs.org/articles/94/i15/Five-chemists-should-won-Nobel.html.

3 The three laureates named the discovered structure “buckminsterfullerene”.

4 The following query was used: ts=(“bet equation” OR “Brunauer Emmett Teller equation”) AND DOCUMENT TYPES: (Article OR Review) Indexes=SCI-EXPANDED, Timespan=All years.

5 A Nobel Prize lecture can appear in more than one publication. If a lecture publication was among the top three retrieved publications, it was disregarded.

6
http://www.kth.se/bibliometrics/classification/2015Q3/.

7 For each seed publication, its three related publications belong to the same class at level 2.

8 We use T11, and not T12, as the endpoint in these comparisons, due to incomplete publication data for the year 2015. Cf. what we write in the first paragraph of the “Results” section.
